# A Serum MicroRNA Signature Is Associated with the Immune Control of Chronic Hepatitis B Virus Infection

**DOI:** 10.1371/journal.pone.0110782

**Published:** 2014-10-28

**Authors:** Maurizia Rossana Brunetto, Daniela Cavallone, Filippo Oliveri, Francesco Moriconi, Piero Colombatto, Barbara Coco, Pietro Ciccorossi, Carlotta Rastelli, Veronica Romagnoli, Beatrice Cherubini, Maria Wrang Teilum, Thorarinn Blondal, Ferruccio Bonino

**Affiliations:** 1 Laboratory of Molecular Genetics and Pathology of Hepatitis Viruses, Hepatology Unit, Reference Center of the Tuscany Region for Chronic Liver Disease and Cancer, University Hospital of Pisa, Pisa, Italy; 2 Division of Dx and Services, Exiqon A/S, Copenhagen, Denmark; 3 Digestive and Liver Disease, General Medicine II Unit, University Hospital of Pisa, Pisa, Italy; CRCL-INSERM, France

## Abstract

**Background and Aims:**

The virus/host interplay mediates liver pathology in chronic HBV infection. MiRNAs play a pivotal role in virus/host interactions and are detected in both serum and HBsAg-particles, but studies of their dynamics during chronic infection and antiviral therapy are missing. We studied serum miRNAs during different phases of chronic HBV infection and antiviral treatment.

**Methods:**

MiRNAs were profiled by miRCURY-LNA-Universal-RT-miRNA-PCR (Exiqon-A/S) and qPCR-panels-I/II-739-miRNA-assays and single-RT-q-PCRs. Two cohorts of well-characterized HBsAg-carriers were studied (median follow-up 34–52 months): a) training-panel (141 sera) and HBsAg-particles (32 samples) from 61 HBsAg-carriers and b) validation-panel (136 sera) from 84 carriers.

**Results:**

Thirty-one miRNAs were differentially expressed in inactive-carriers (IC) and chronic-hepatitis-B (CHB) with the largest difference for miR-122-5p, miR-99a-5p and miR-192-5p (liver-specific-miRNAs), over-expressed in both sera and HBsAg-particles of CHB (ANOVA/U-test p-values: <0.000001/0.000001; <0.000001/0.000003; <0.000001/0.000005, respectively) and significantly down-regulated during- and after-treatment in sustained-virological-responders (SVR). MiRNA-profiles of IC and SVR clustered in the heatmap. Liver-miRNAs were combined with miR-335, miR-126 and miR-320a (internal controls) to build a MiR-B-Index with 100% sensitivity, 83.3% and 92.5% specificity (−1.7 cut-off) in both training and validation cohorts to identify IC. MiR-B-Index (−5.72, −20.43/14.38) correlated with ALT (49, 10/2056 U/l, *ρ* = −0.497, p<0.001), HBV-DNA (4.58, undetectable/>8.3 Log_10_ IU/mL, *ρ* = −0.732, p<0.001) and HBsAg (3.40, 0.11/5.49 Log_10_ IU/mL, *ρ* = −0.883, p<0.001). At multivariate analysis HBV-DNA (p = 0.002), HBsAg (p<0.001) and infection-phase (p<0.001), but not ALT (p = 0.360) correlated with MiR-B-Index. In SVR to Peg-IFN/NUCs MiR-B-Index improved during-therapy and post-treatment reaching IC-like values (5.32, −1.65/10.91 vs 6.68, 0.54/9.53, p = 0.324) beckoning sustained HBV-immune-control earlier than HBsAg-decline.

**Conclusions:**

Serum miRNA profile change dynamically during the different phases of chronic HBV infection. We identified a miRNA signature associated with both natural-occurring and therapy-induced immune control of HBV infection. The MiR-B-Index might be a useful biomarker for the early identification of the sustained switch from CHB to inactive HBV-infection in patients treated with antivirals.

## Introduction

Hepatitis B Virus (HBV) is not cytopathic and liver pathology is mediated by the interplay between virus and immune system; accordingly, 3 major phases are identified in chronic HBV infection: immune tolerance, activation and immune control [1]–[3]. High viral load and circulating hepatitis B “e” antigen (HBeAg) in absence of virus induced liver disease characterize the immune tolerance phase, that is lost when the antiviral immune reaction tries to taper HBV replication causing liver inflammation, namely HBeAg positive chronic hepatitis B (CHB) [1]–[3]. An effective immune activation leads to the immune control of HBV replication (HBV-DNA<2000 IU/mL) and HBeAg/anti-HBe seroconversion, that identifies the inactive HBeAg-negative, HBsAg carrier (IC). When the control of viral replication is ineffective HBeAg-defective HBV-variants are selected with progression to HBeAg-negative, anti-HBe-positive CHB, the most prevalent form of HBV disease worldwide [4]–[6]. Antiviral therapy is aimed to halt disease progression suppressing viral replication with indefinite nucleos(t)ide analogs (NUCs) treatment or achieving a sustained off-therapy immune control after finite courses of Pegylated-interferon (Peg-IFN) [1]–[3]. In recent years monitoring of HBV-DNA and HBsAg serum levels significantly improved the management of antiviral treatment [7]–[9]. However, the decline of HBV-DNA serum levels during Peg-IFN therapy does not help to distinguish responders (SVR) from relapsers (REL) and early HBsAg kinetics are predictive of response in Peg-IFN, but non in NUC treated patients [8]. In addition the kinetics of constitutive HBV markers are the biological hallmark of viral expression, but not the expression of the host's response to antivirals. Thus, serum biomarkers of the effective control of HBV infection are currently unsatisfactory and remain an unmet need in the clinical management of chronic HBV carriers [1]–[3]. MicroRNAs (miRNAs), are small endogenous single-stranded RNAs that modulate the expression of cellular genes and play key roles in vital biological processes and immunity [10]. Host- and/or virus-encoded miRNAs appear to regulate the outcome of both infections and diseases [11]–[12], as shown for the liver-specific miRNA, miR-122, that is essential for the replication of hepatitis C virus (HCV) [13]. In chronic HBV infection several miRNAs are up-regulated in the serum of HBsAg carriers as compared to controls and circulating HBsAg particles carry specific hepatocellular miRNAs [14]–[15]. Preliminary reports suggest that serum miRNA profiling may contribute to characterize chronic HBV carriers with or without HCC [16]–[18]. However, studies focused on the relations between serum miRNAs and the different phases of chronic HBV infection and during antiviral therapy are missing: in the present study we analysed the dynamics of miRNA profiles in sera and circulating HBsAg particles of IC and CHB according to treatment response.

## Patients and Methods

### Study population

#### Training cohort

Serum samples (141) were obtained from 61 (40 males, median age 50 years, 21–79) well characterized HBsAg carriers, 57 infected with HBV genotype D and 4 genotype A (Table 1). In case of low HBV-DNA levels (<20000 IU/mL) and normal transaminases (ALT) at presentation, they were followed for at least 1 year with monthly blood tests for classification [1], [6], thereafter every 3/6 months (m) as all the other HBV carriers. The HBV carriers were followed-up (median follow-up 34, 18–144 m) at the Hepatology Unit of the University Hospital of Pisa. The study was approved by the local Ethic Committee of the University Hospital of Pisa. All patients provided informed written consent.

**Table 1:** Baseline and treatment features of study (A) and validation (B) cohorts.

Cohort	Group	Patients	Sera samples	Age	Gender	ALT	HBsAg	HBV-DNA	Treatment	Response
		n.	n.	years	M/F	U/L	Log_10_ UI/mL	Log_10_ UI/mL		
**A**	**Overall**	61	141	50 (21–79)	40/21	52 (11–558)	3.46 (0.18–5.49)	4.45 (n.d. –>8.23)	29 Untreated, 21 IFN±NUCs, 11 NUCs	*IFN*: 13 SVR, 5 Rel, 3 NR. *NUCs*: 2 SVR, 9 OTR.
	IC	16	16	54 (32–79)	12/4	20 (11–34)	1.58 (0.18–3.22)	1.91 (n.d. –3.10)	No	
	HDV	4	4	38 (25–59)	3/1	179 (86–207)	4.25 (4.17–4.39)	1.76 (n.d. –1.98)	No	
	HBeAg pos carriers	5	5	36 (21–71)	3/2	64 (26–84)	4.84 (3.71–5.49)	7.88 (7.06–>8.23)	No	
	HBeAg neg CHB	36	116	49 (33–62)	22/14	57 (17–558)	3.57 (1.13–4.09)	4.94 (n.d. –8.12)	4 Untreated, 21 IFN±NUCs, 11 NUCs	*IFN*: 13 SVR, 5 Rel, 3 NR. *NUCs*: 2 SVR, 9 OTR.
**B**	**Overall**	84	136	54 (17–79)	59/25	48 (10–2056)	3.38 (0.11–5.49)	4.87 (n.d. –>8.23)	32 Untreated, 15 IFN±NUCs, 37 NUCs	*IFN*: 6 SVR, 4 Rel, 5 NR. *NUCs*: 30 OTR.
	IC	23	23	55 (26–74)	16/7	22 (10–42)	1.43 (0.11–3.14)	1.67 (n.d. –3.13)	No	
	HBeAg pos IT	5	5	27 (19–46)	4/1	27 (19–41)	4.60 (4.41–5.49)	>8.23	No	
	HBeAg pos CHB	16	32	45 (22–67)	14/2	81 (21–374)	4.58 (2.92–5.22)	8.04 (4.53 –>8.23)	9 IFN±NUCs, 7 NUCs,	*IFN*: 5 SVR, 4 NR. *NUCs*: 4 SC, 3 OTR.
	HBeAg neg CHB	40	76	55 (17–79)	25/15	101 (13–2056)	3.47 (2.57–4.56)	5.23 (n.d. –>8.23)	4 Untreated, 6 IFN±NUCs, 30 NUCs	*IFN*: 1 SVR, 4 Rel, 1 NR *NUCs*: 30 OTR

n.d.  =  not detectable, SVR = Sustained virologic response, SC = HBeAg to anti-HBe seroconversion, Rel = relapse, NR = no response, OTR = on treatment response.

HBsAg carriers were classified according to their biochemical and viral profiles [1], [6]: a) 5 HBeAg positive carriers [1 immune tolerant, IT (HBV-DNA>8.3 log_10_ IU/mL, HBsAg>4.39 log_10_ IU/mL, normal ALT) and 4 with chronic hepatitis B]; b) 16 IC (HBV-DNA persistently <2000 IU/mL and normal ALT); c) 36 HBeAg negative CHB patients [HBV-DNA>2000 IU/mL with evidence of HBV induced liver disease (at histology and/or liver elastometry and ultrasound)]; d) 4 HBeAg negative, anti-HDV (Hepatitis D Virus) positive carriers with chronic hepatitis (IgM anti-HDV and HDV-RNA positive).

Thirty-two HBeAg negative CHB patients underwent to antiviral treatment: 21 received Peg-IFN 180 µg/week ± NUCs for 12–36 m and 11 NUCs for a median period of 60 m (36–114 m); in 2 patients who cleared HBsAg, NUCs were discontinued after 36 and 112 m, respectively. Response to Peg-IFN was defined as: a) end of treatment (EOT) response if HBV-DNA was <2000 IU/mL at EOT (18 cases); b) non response (NR) if HBV-DNA was >2000 IU/mL at EOT (3 cases); c) relapse (REL) when florid viral replication recurred after an EOT response (5 cases); d) sustained virologic response (SVR) if viral load persisted <2000 IU/mL at every 3 month controls for at least 12 months after EOT (13 cases). Sera were obtained at baseline (BL) and week 24 post-treatment (post-T-FU) in all Peg-IFN treated patients; additional sampling (week 12 and 24 during treatment, EOT) were obtained in 14 patients. In NUCs treated patients (all achieved undetectable on treatment serum HBV-DNA) serum samples were obtained at BL and at their last available on treatment follow-up (EOF) or 24 weeks after NUCs discontinuation (post-T-FU). In 5 HBeAg positive carriers, 16 IC carriers and 4 HBV/HDV patients sera were obtained at a single time point; in 4 HBeAg negative CHB patients with fluctuating disease profiles the sampling was performed during spontaneous remission (ALT range 19–28 U/L; HBV-DNA 3.71–4.57 Log_10_ IU/mL) and at the time of HBV reactivation (ALT range 210–550 U/L; HBV-DNA 4.35–7.18 Log_10_ IU/mL). MiRNA profiling was obtained from whole serum in all samples; in addition, to target specifically hepatocellular miRNAs, the profiling was performed in HBsAg immune precipitated (IP) particle from 32 sera [6 from IC and 26 from 13 HBeAg negative CHB patients, with SVR (5), REL (5) or NR (3) to Peg-IFN].

### Validation cohort

A second well characterized cohort of 84 HBV carriers (59 males, median age 54 years, 17–79) followed-up for 52 m (18–159 m) and classified according to the above defined criteria was used to generate the validation serum panel (overall 136 sera) of the MiR-B-Index (Table 1). Serum samples were obtained at a single point in 23 IC, 5 IT and 4 untreated HBeAg negative CHB patients. In 52 (16 HBeAg positive) treated patients, samples were collected at BL and 24 week Post-T-FU in the 15 Peg-IFN ± NUC treated patients [9 HBeAg positive: 5 with SVR (HBeAg/anti-HBe seroconversion and HBV-DNA<2000 IU/mL at EOT and in post-T-FU); 6 HBeAg negative: 1 SVR, 4 REL, 1 NR] and at BL and EOF in the 37 patients (7 HBeAg positive) treated with NUCs (all achieved undetectable on treatment HBV-DNA, none cleared HBsAg). In addition, 15 serum samples of the study cohort were retested together with the validation cohort.

### Serological tests

HBsAg qualitative and quantitative, anti-HBs, anti-HBc, IgM anti-HBc HBeAg and anti-HBe, anti-HCV, anti-HDV and anti-HIV were detected by commercially available immunoassays (Abbott laboratories, N. Chicago, IL, USA). Serum HBV DNA levels were quantified by COBAS TaqMan assay, sensitivity 6 IU/mL, dynamic range 6–1.70×10^8^ IU/mL (Roche Diagnostic Systems Inc, Mannheim, Germany). HBV genotyping was performed by direct sequencing of the small HBs region. Serum ALT levels were tested by routine biochemistry (normal range: <45 U/L and <33 U/L for male and female respectively). Circulating HBsAg particles were obtained by immunoprecipitation as previously reported [14].

### RNA-isolation, cDNA-synthesis and RT-q-PCR

Total RNA was extracted from 200 µL serum or HBsAg-IP (re-suspended in PBS) using miRNeasy-Mini-kit (Qiagen-Inc.) as described [19]. RNA (16 µL) was reverse-transcribed and profiled on q-PCR panels in 80 µL reactions using the miRCURY-LNA Universal-RT-cDNA-Synthesis and RT-miRNA-PCR (Exiqon-A/S). The cDNA was diluted 1∶50 and assayed in 10 µL PCR reactions; each miRNA was assayed once by qPCR on the Ready-to-use microRNA-PCR panels I/II containing 739-miRNA-assays. The amplification was performed in a LightCycler 480-System (Roche-Applied-Science) in 384-well-plates.

Single RT-q-PCR for each of the 6 miRNAs (miR-122-5p,miR-99a-5p, miR-192-5p, miR-126-3p, miR-335-5p and miR-320a) were used for the MiR-B-Index evaluation. Briefly, total RNA was extracted from 200 µL serum using miRCURY RNA Isolation kit (Exiqon). RNA (2 µL) was reverse-transcribed using the miRCURY-LNA Universal-RT-cDNA-Synthesis. The cDNA was diluted 1∶50 and amplified in 10 µL PCR reactions using RT-miRNA-PCR (Exiqon-A/S). The amplification was performed on LightCycler 2.0 System (Roche-Applied-Science) using specific primers to quantify each miRNA.

### Data analysis and statistics

The amplification curves of miRNAs were analyzed using the Roche LC software for the quantification of cycles (Cq), by the second derivative max method, according to the recommendations of the Minimum Information for Publication of Quantitative Real-time PCR Experiments (MIQE) [20] and for the melting curve analysis. The amplification efficiency was calculated using the LinReg algorithm with criteria between 0.8–1.1 [21]–[22]. All assays were inspected for distinct melting curves and the Tm was checked to be within known specifications for each particular assay. Furthermore any sample assay data point to be included in the data analysis had to be detected with 5 Cq less than the corresponding negative control assay data point and with a Cq<37 for serum samples, but 3 Cq less than the corresponding negative control assay data point and with a Cq<37 for IP HBsAg samples due to lower signal. Data that did not pass these criteria were omitted from any further analysis. Normfinder was used to find the best normalizer candidates which in this study was the Global Mean of all assays expressed in all samples tested [23]–[24]. By this approach we normalised all miRNAs Cq, obtaining for each miRNA the ΔCq. In all statistical comparison we used a number of assays based on the criteria that the smallest groups of analysed cases needed data present in all samples. We subtracted the microRNAs value from the sample average (global mean), thus the larger the value, the higher is the miRNA expression in reported results. For example when we obtain a positive ΔΔCq value after subtraction, a control group mean value from an affected group mean value 2^(ΔCq(affected)−ΔCq(Ctrl))^ then a value of one means 2^(1)^, namely double as much expression for the given miRNA, on average, in the affected group. Statistics and data presentation were performed in Microsoft Excel, GraphPad Prism 6.03 and TIGR’s Multiple Experiment Viewer (MEV) version 4.8 [25]–[26]. Correlations of miRNA profiling, MiR-B-Index and their variation over time in the different groups of HBsAg carriers were analysed by Spearman test, Mann-Whitney U-Test, Kruskal-Wallis test, Student t test, analysis of variance (ANOVA) and ANOVA for repeated measures, when appropriate. When values per group were few for ANOVA or other statistics we used the fold-change-method to evaluate up- or down-regulations using a 2-fold-change as threshold [27]. Linear regression analysis was performed to evaluate the independent variables associated with MiR-B-Index and its Δ-variation. The diagnostic performance of Mir-B-Index for the identification of IC versus CHB patients was evaluated by ROC curve analysis; the selection of cut-off value was aimed to maximize the sensitivity (100% of IC). The statistical analyses were performed using SPSS version 19.0 (IBM Corp., Armonk, NY). The statistical significance was defined as p<0.05, after Bonferroni correction when required.

## Results

### HBsAg particles miRNA profiling

**Figure 1:**
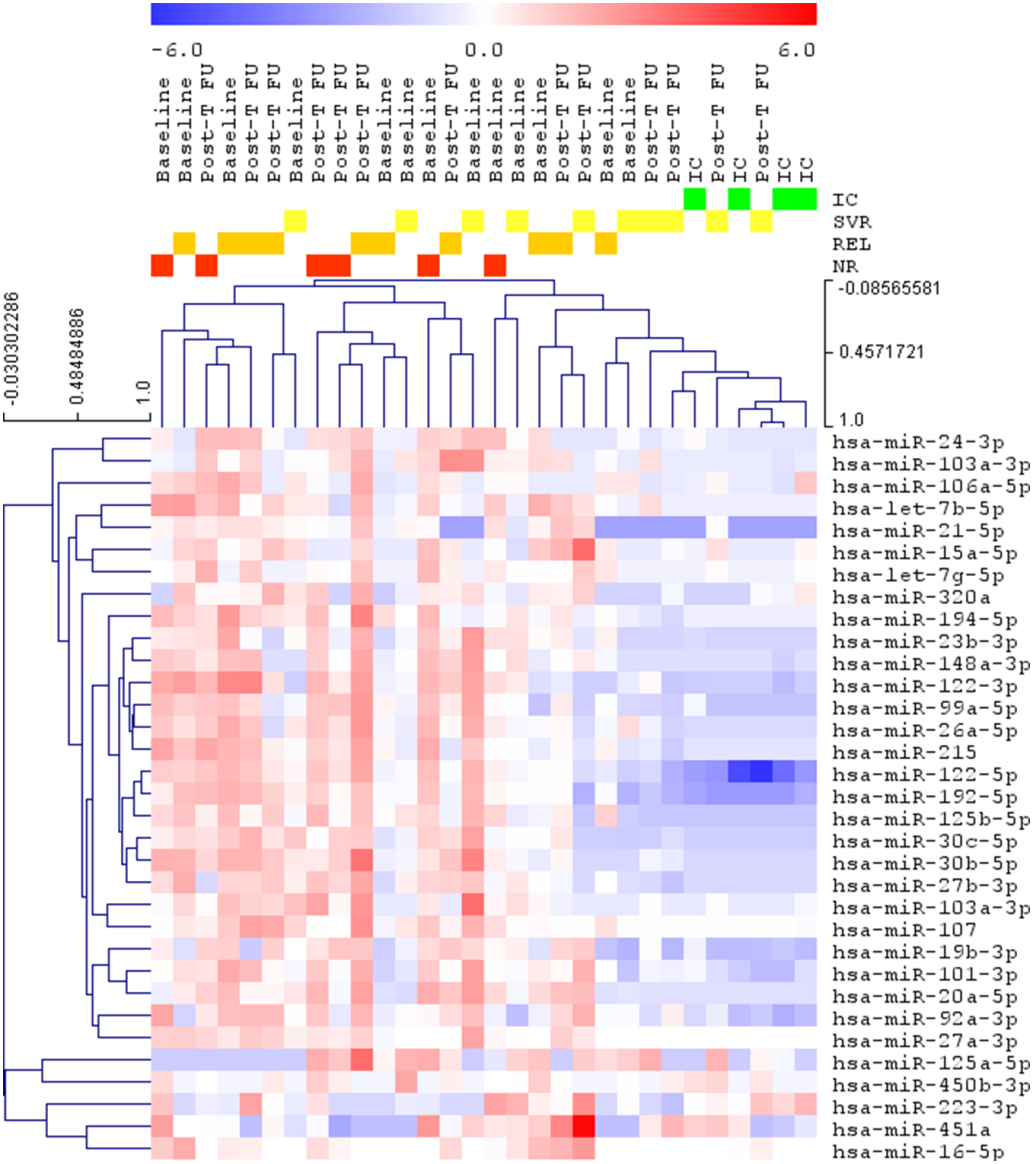
Hierarchical clustering of miRNAs from HBsAg particles and samples. The heatmap shows the result of the two-way hierarchical clustering of miRNAs from HBsAg particles and samples (zero centered). The colour scale shown at the top illustrates the relative expression level of a miRNA across all samples: red colour represents an expression level above mean, blue colour represents expression lower than the mean. The SVR post-T FU (Peg-IFN treated patients) group clusters with the IC group.

A 44 miRNAs average yield was obtained from each HBsAg-pellet with an overall mean Cq of 34 after quality control (QC) and background filtering. No miRNA was expressed in all samples, therefore, normalization was performed adding the UniSp4 spike-in (RNA-spike-in-kit, Exiqon-AS) in the RNA purification process to monitor small RNA yields. When comparing the different groups (IC, untreated and treated HBeAg-negative CHB patients), we analyzed 32 miRNAs with 15 or more values across the 32 samples (37 Cq background filter value was given for missing or filtered values). Differences in the miRNAs expression (ΔCq) were observed comparing IC samples with that of BL-CHB (SVR, REL, NR) and REL/NR-post-T-FU, but not between IC and SVR-post-T-FU ones, that showed major difference with the other groups (Table 2, Figure 1). The most differentially expressed miRNAs (ΔΔCq) comparing SVR-BL-CHB to both SVR-post-T-FU and IC were miR-122-5p (ΔΔCq 2.77 and 3.34), miR-192-5p (ΔΔCq 2.35 and 2.04), miR-125b-5p (ΔΔCq 1.71 and 1.56), miR-30c-5p (ΔΔCq 1.66 and 0.92) and miR-99a-5p (ΔΔCq 1.57 and 1.30). The same miRNAs were up-regulated in all BL-CHB and REL/NR-post-T-FU. Let-7g-5p (ΔΔCq −0.33 and −0.96), miR-223-3p (ΔΔCq −0.36 and −2.22), miR-16-5p (ΔΔCq −0.43 and −1.21), miR-15a-5p (ΔΔCq −0.98 and −1.37) and miR-451a (ΔΔCq −2.07 and −1.91) were down-regulated. Differences were more evident (Table 2; Figure 1) after compiling the miRNA data of BL-CHB with REL/NR-post-T-FU and comparing them with IC and/or SVR-post-T-FU. Overall 18 miRNAs were over-expressed (ΔΔCq>1) in all BL-CHB and in REL/NR-post-T-FU samples when compared to SVR-Post-T-FU, and 12 miRNAs when compared to IC. Only 1 and 2 miRNAs were down-regulated (ΔΔCq<−1) after comparison with SVR-post-T-FU and IC respectively. MiR-122-5p, miR-192-5p, miR-125b-5p and miR-99a-5p showed the highest up-regulation; miR-451a was down regulated in 1 of the 2 comparisons (Table 2). An unsupervised two-way hierarchical clustering of miRNAs and samples showed the clustering of IC with SVR-post-T-FU (Figure 1). Finally, we compared the most differentially expressed miRNAs (32) in ours and in the study of Novellino *et al*. [14] (31 miRNAs): in spite of the different platforms which were used, 9 miRNAs were consistently detected in both series, miR-19b, miR-24, miR-26a, miR-27a, miR-30b, miR-30c, miR-106b, miR-122, miR-223.

**Table 2:** Mean ΔCq values (± standard deviation) of miRNAs from HBsAg particles per groups in Peg-IFN treated patients and inactive carriers [NR-BL (A); NR-Post-T-FU (B); REL BL (C); REL-Post-T-FU (D); SVR BL (E); SVR-Post-T-FU (F); IC (G); NR, REL, SVR BL and REL/NR Post-T-FU (H)].

Assay	ΔCq (mean ± SD) per group								Group comparisons (ΔΔ Cq)			
	A	B	C	D	E	F	G	H	F-E	G-E	F-H	G-H
	NR	NR	REL	REL	SVR	SVR	IC	A+B+C+D+E				
	Baseline	Follow up	Baseline	Follow up	Baseline	Follow up						
hsa-miR-122-5p	29.91±0.58	29.78±0.36	30.55±0.98	30.14±0.90	30.89±1.14	33.67±2.05	34.23±1.66	30.33±0.91	2.77	3.34	3.34	3.90
hsa-miR-192-5p	33.38±0.62	33.10±0.72	33.46±0.97	33.30±0.86	34.10±1.41	36.44±0.56	36.14±1.41	33.51±0.97	2.35	2.04	2.93	2.63
hsa-miR-125b-5p	34.41±0.04	34.70±0.90	34.59±0.81	34.78±0.72	35.29±1.21	37.00±0.00	36.86±0.32	34.79±0.84	1.71	1.56	2.21	2.06
hsa-miR-122-3p	34.12±0.62	34.32±0.67	35.14±1.93	34.31±1.35	35.53±1.31	36.74±0.73	36.95±0.55	34.77±1.36	1.21	1.42	1.97	2.18
hsa-miR-99a-5p	34.23±0.80	33.90±0.05	35.03±1.24	34.58±1.13	35.19±1.20	36.76±0.23	36.49±0.72	34.69±1.07	1.57	1.30	2.07	1.80
hsa-miR-30c-5p	35.15±0.66	34.99±0.55	35.63±1.12	34.58±0.50	35.26±1.44	36.92±0.19	36.18±1.84	35.13±0.96	1.66	0.92	1.79	1.05
hsa-miR-27b-3p	35.35±0.68	35.49±1.32	35.54±1.14	34.87±0.83	35.56±0.83	37.12±0.27	36.21±1.76	35.35±0.91	1.56	0.66	1.77	0.86
hsa-miR-23b-3p	34.91±0.43	35.28±0.86	35.39±1.16	35.52±1.11	35.43±1.33	36.90±0.20	36.30±1.47	35.35±1.00	1.47	0.86	1.56	0.95
hsa-miR-215	34.86±1.21	34.96±0.73	35.60±0.93	35.52±1.16	36.23±0.82	37.09±0.35	37.00±0.00	35.53±1.01	0.86	0.77	1.56	1.47
hsa-miR-30b-5p	34.89±0.76	34.99±0.31	35.59±1.38	34.29±1.11	35.50±1.53	36.73±0.38	36.04±2.16	35.07±1.20	1.23	0.54	1.66	0.96
hsa-miR-26a-5p	34.78±0.62	34.84±0.34	35.50±0.95	35.32±1.32	35.41±0.96	36.90±0.34	35.84±2.59	35.24±0.92	1.49	0.43	1.66	0.60
hsa-miR-148a-3p	35.10±0.82	35.47±0.57	35.80±0.96	35.31±1.21	35.80±1.36	36.71±0.64	36.69±0.91	35.53±1.01	0.91	0.89	1.18	1.16
hsa-miR-194-5p	35.49±0.98	35.79±0.76	35.70±1.02	35.50±1.17	36.50±0.52	37.18±0.29	37.00±0.00	35.83±0.92	0.68	0.50	1.36	1.17
hsa-miR-21-5p	33.87±0.33	34.16±0.37	34.52±1.46	34.03±1.71	35.44±1.48	35.72±1.80	35.55±3.24	34.48±1.35	0.28	0.11	1.24	1.07
hsa-miR-19b-3p	34.57±0.34	34.04±0.35	35.80±1.13	34.41±1.22	35.32±1.19	35.98±1.48	35.24±2.95	34.93±1.14	0.66	−0.08	1.06	0.32
hsa-miR-92a-3p	34.47±1.30	34.71±0.99	35.26±1.01	33.81±0.42	35.17±1.18	35.29±1.33	35.56±3.05	34.70±1.06	0.12	0.40	0.58	0.86
hsa-miR-20a-5p	35.31±1.21	35.24±0.98	35.87±1.10	35.01±0.86	35.93±1.33	36.50±1.09	35.79±2.69	35.51±1.07	0.57	−0.13	0.99	0.28
hsa-miR-101-3p	34.64±0.70	34.45±0.72	34.66±1.20	34.29±0.89	35.18±1.45	35.42±1.39	35.26±2.25	34.66±1.04	0.24	0.08	0.76	0.60
hsa-miR-103a-3p	36.08±0.61	35.73±0.64	36.50±0.59	35.33±1.18	35.86±1.20	36.74±0.58	35.84±2.74	35.90±0.94	0.88	−0.02	0.84	−0.06
hsa-miR-125a-5p	35.63±1.76	34.95±1.80	35.63±1.31	35.61±2.15	35.34±1.54	34.83±1.38	36.60±0.54	35.46±1.56	−0.51	1.26	−0.63	1.14
hsa-miR-24-3p	35.22±0.55	35.19±0.40	36.15±1.04	35.64±0.92	36.19±0.88	36.89±0.15	35.61±2.66	35.77±0.88	0.70	−0.58	1.12	−0.16
hsa-miR-107	36.26±0.68	36.85±0.23	36.50±0.59	35.57±1.16	36.30±0.79	37.00±0.01	36.01±2.25	36.25±0.84	0.71	−0.29	0.76	−0.24
hsa-miR-450b-3p	36.51±0.62	36.19±0.60	36.71±0.65	36.64±0.36	36.41±0.99	36.44±0.55	36.79±0.47	36.52±0.64	0.02	0.38	−0.08	0.27
hsa-miR-106a-5p	35.82±0.42	36.11±1.12	35.94±1.16	35.92±1.13	36.37±0.61	36.88±0.38	35.58±2.38	36.04±0.88	0.51	−0.79	0.83	−0.47
hsa-let-7b-5p	35.04±0.60	36.33±1.25	35.49±1.28	35.76±0.78	36.77±0.41	36.51±0.67	36.16±1.88	35.91±1.02	−0.26	−0.61	0.60	0.25
hsa-miR-27a-3p	36.56±0.60	36.31±0.27	36.69±0.57	36.09±0.51	36.50±0.92	36.89±0.26	35.87±2.52	36.43±0.62	0.38	−0.63	0.46	−0.56
hsa-miR-320a	36.07±0.89	35.70±1.24	35.89±1.20	34.79±0.82	35.93±0.91	36.09±0.99	35.29±2.40	35.64±1.03	0.16	−0.63	0.45	−0.34
hsa-let-7g-5p	35.89±0.83	35.95±1.02	36.56±0.46	36.10±0.76	36.92±0.18	36.59±0.69	35.96±2.15	36.35±0.71	−0.33	−0.96	0.23	−0.39
hsa-miR-16-5p	36.63±0.72	36.63±0.42	36.20±0.90	36.39±0.74	36.87±0.28	36.44±1.04	35.66±3.00	36.53±0.65	−0.43	−1.21	−0.09	−0.87
hsa-miR-15a-5p	36.00±0.98	36.41±1.03	35.95±0.75	35.39±0.56	36.79±0.70	35.81±1.71	35.42±2.95	36.09±0.86	−0.98	−1.37	−0.28	−0.67
hsa-miR-223-3p	34.90±1.42	36.16±0.93	36.59±0.66	35.86±1.35	35.94±1.12	35.58±1.80	33.73±3.41	35.96±1.13	−0.36	−2.22	−0.38	−2.23
hsa-miR-451a	33.40±1.04	36.05±1.43	35.66±1.31	35.31±1.92	35.71±1.40	33.63±2.79	33.79±3.50	35.32±1.58	−2.07	−1.91	−1.69	−1.53

On the right comparisons between groups F and E; G and E; F and H; G and H.

### Serum miRNA profiling

In total we profiled 141 whole sera from 61 chronic carriers in different phases of HBV infection and at different time points during antiviral therapy. The overall miRNA yields were good, 152 of the 739 tested miRNAs passed background filtering and QC and 26 miRNAs were expressed in all samples after excluding only two samples because of low yields (<50 miRNAs passing filtering and QC). Of those 26 microRNAs 7, 14 and 5 assays show standard deviation <0.5, 0.5–1.0 and >1.0 respectively, after normalization. Sixty-six of the 152 miRNAs that passed the QC criteria were expressed in all samples of the smallest groups (HDV, n = 4 and HBeAg Positive, n = 5), were used in the analysis. The dataset was normalized using the global mean (n = 26 assays) method [24].

#### Untreated HBV Carriers

One way ANOVA (after Bonferroni correction, cut-off p = 0.000758) revealed 31 miRNAs with significantly different expression: the largest differences were primarily observed between IC and both HBeAg positive and negative CHB, secondly between HBeAg-positive and HBeAg-negative CHB (Table 3). Among HBeAg positive carriers the miRNA profiling of one immune tolerant carrier was comparable to those of the other 4 patients with CHB. The statistical power of comparisons between HBeAg-positive CHB and HDV/CHB was weakened by the small sample size. Since several miRNAs from HBeAg-negative-CHB failed the Shapiro-Wilk normality test (data not shown), we used individual Wilcoxon, Mann-Whitney U tests yielding very similar results (Table 3). Significant differences were found between IC and CHB patients and the most significant ones affected the same miRNAs showing the largest difference in HBsAg particles of CHB patients and IC, namely miRNA-122-5p (ANOVA and U test p-values: <0.000001 and 0.000001 respectively); miRNA-192-5p (ANOVA and U test p-values: <0.000001 and 0.000005); miRNA-99a-5p (ANOVA and U test p-values: <0.000001 and 0.000003). Other miRNAs with the largest differences were miR-148a-3p (ANOVA and U test p-values: <0.000001 and 0.000009) and miR-126-3p (ANOVA and U test p-values: <0.000001 and 0.003751). Overall CHB patients showed significantly more liver specific miRNAs than IC. The IC miRNA profile correlated with that of subjects without liver disease of Wang et al. [28] who used the miRCURY qPCR platform [correlations between (n = 47 assays) mean un-normalized-ΔCq values (40-Cq) Pearson-*r* = .9470, *r* ^2^ = .8968, p<0.000000001; Spearman-*ρ* = 0.9373, p<0.000000001, data not shown].

**Table 3:** One-way ANOVA and Wilcoxon Mann Whitney U test in 61 untreated HBV carriers: 31 miRNAs (out of 66 tested) showed significant differential expression after either ANOVA or the U test on the four group comparison (Bonferroni multiple testing cut-off = 0.000758).

Assay	ANOVA					Wilcoxon Mann-Whitney U test					
	ΔCq (mean ± SD) per group				p value	p value					
	HBeAg Ne CHB	HDV	IC	HBeAg pos CHB		IC	HDV	HBeAg pos	HDV	HBeAg pos	HDV
						vs	vs	vs	vs	vs	vs
						HBeAg Neg	HBeAg Neg	HBeAg Neg	IC	IC	HBeAg pos
hsa-miR-122-5p	3.87±1.81	3.21±2.24	0.33±1.16	5.81±0.39	<0.000001	0.000001	0.429017	0.001182	0.035729	0.001063	0.014306
hsa-miR-148a-3p	−0.66±0.95	−0.92±0.99	−2.47±0.79	1.01±0.59	<0.000001	0.000009	0.557818	0.000708	0.031450	0.001357	0.014306
hsa-miR-192-5p	0.18±1.71	0.04±2.03	−3.33±1.60	2.39±0.46	<0.000001	0.000005	0.895133	0.000781	0.019472	0.001194	0.014306
hsa-miR-126-3p	1.03±0.60	0.88±0.15	1.66±0.61	−0.78±0.92	<0.000001	0.003751	0.455102	0.000588	0.016395	0.001063	0.014306
hsa-miR-99a-5p	−0.33±1.60	−0.95±2.11	−3.05±0.85	1.66±0.33	<0.000001	0.000003	0.470703	0.001250	0.071861	0.001063	0.014306
hsa-miR-142-3p	0.80±0.77	0.86±0.31	1.57±0.65	−1.11±0.80	<0.000001	0.001467	0.792069	0.001182	0.045500	0.001063	0.014306
hsa-miR-142-5p	−3.82±0.98	−3.86±0.50	−2.58±0.86	−5.92±0.92	<0.000001	0.000322	0.835705	0.001260	0.021448	0.001063	0.014306
hsa-miR-223-3p	2.01±0.95	2.63±0.82	3.06±0.38	0.05±1.15	<0.000001	0.000051	0.173178	0.004865	0.317311	0.001063	0.027486
hsa-miR-194-5p	−1.46±1.68	−1.59±1.90	−4.39±1.32	1.09±0.59	<0.000001	0.000074	0.758415	0.000509	0.036714	0.001837	0.014306
hsa-miR-424-5p	−1.61±0.78	−1.56±0.40	−0.84±0.33	−3.77±1.42	<0.000001	0.004321	0.958650	0.002351	0.016210	0.002200	0.014306
hsa-miR-215	−1.39±1.80	−1.58±2.15	−4.39±1.28	1.09±0.47	<0.000001	0.000067	0.929976	0.000898	0.026453	0.001837	0.014306
hsa-miR-378a-3p	−1.81±0.93	−2.20±1.22	−3.26±0.58	−0.63±0.42	0.000001	0.000026	0.475551	0.004282	0.141032	0.001357	0.027486
hsa-miR-30b-5p	−0.42±0.87	−0.44±0.63	−1.07±0.53	1.40±0.37	0.000001	0.004263	0.895133	0.000380	0.109599	0.001063	0.014306
hsa-miR-15b-5p	−1.31±0.82	−1.27±0.63	−0.40±0.41	−2.51±0.32	0.000002	0.000168	0.928155	0.003633	0.021448	0.001063	0.014306
hsa-miR-23a-3p	−0.41±0.57	0.17±0.33	0.37±0.41	−1.20±0.71	0.000004	0.000033	0.030464	0.065087	0.317311	0.007686	0.033895
hsa-miR-27b-3p	−0.50±0.77	−0.52±0.70	−1.25±0.39	0.80±0.40	0.000004	0.000220	0.964041	0.001087	0.071015	0.001194	0.014306
hsa-miR-23b-3p	−0.56±1.23	−1.01±1.02	−1.73±0.44	1.24±0.63	0.000008	0.000393	0.379537	0.001549	0.133614	0.001063	0.014306
hsa-miR-365a-3p	−3.15±1.48	−4.11±2.39	−5.47±0.61	−1.51±0.60	0.000011	0.000228	0.354539	0.007391	0.157299	0.002200	0.014306
hsa-miR-26b-5p	−1.49±0.57	−1.60±0.47	−1.89±0.62	−0.22±0.51	0.000012	0.026989	0.792069	0.000781	0.317311	0.001944	0.014306
hsa-miR-122-3p	−1.05±2.00	−1.52±2.02	−4.45±1.80	1.80±0.45	0.000012	0.000785	0.458902	0.000638	0.058782	0.004483	0.014306
hsa-miR-34a-5p	−3.22±1.53	−3.96±2.10	−5.62±0.71	−3.19±1.60	0.000190	0.000023	0.598020	0.861255	0.117185	0.001837	0.806496
hsa-miR-30c-5p	−0.42±1.01	−0.72±0.86	−1.19±0.51	1.22±0.37	0.000025	0.002531	0.356170	0.001031	0.161513	0.001063	0.014306
hsa-let-7i-5p	−3.07±1.02	−2.88±0.84	−2.26±0.54	−4.64±0.52	0.000038	0.001100	0.724571	0.003881	0.230139	0.001063	0.014306
hsa-miR-30d-5p	−4.42±0.47	−4.21±0.15	−4.72±0.46	−3.45±0.53	0.000043	0.093959	0.186630	0.003558	0.071015	0.004104	0.086411
hsa-miR-93-5p	−1.05±0.78	−0.58±0.93	−0.36±0.48	−1.54±0.13	0.002882	0.000051	0.235499	0.020837	0.548506	0.001063	0.141645
hsa-miR-16-5p	0.95±1.27	1.73±1.08	2.12±0.77	−0.81±1.25	0.000058	0.000771	0.202597	0.015205	0.271332	0.001063	0.014306
hsa-miR-30e-3p	−3.75±1.17	−4.15±0.69	−5.05±1.23	−2.21±0.25	0.000067	0.000654	0.211352	0.000859	0.111164	0.001194	0.014306
hsa-miR-21-5p	2.28±0.50	2.41±0.70	1.56±0.59	2.13±0.30	0.000345	0.000165	0.598020	0.521622	0.057433	0.026026	0.462433
hsa-miR-103a-3p	0.23±0.37	0.10±0.15	0.70±0.34	0.19±0.78	0.001603	0.000210	0.660390	0.268329	0.009322	0.073553	0.624206
hsa-miR-152	−3.68±0.64	−4.38±0.56	−4.44±0.64	−3.42±0.52	0.000682	0.000240	0.034097	0.380758	1.000000	0.007256	0.027486
hsa-miR-15a-5p	1.22±0.98	1.38±0.78	1.84±0.74	−0.29±0.40	0.000304	0.016691	0.792069	0.000898	0.230139	0.001063	0.014306

#### Peg-IFN treated patients

The effect of Peg-IFN on the circulating miRNA profile of HBeAg-negative-CHB was studied comparing BL and week 12 sera in 14 cases [6 SVR, 5 REL and 3 NR]: 8 miRNAs were differentially expressed, but only miR-30e-3p was significantly up-regulated in all patients’ sera after Bonferroni adjustment (ΔΔCq 1.7, p = 0.000354, Table 4). Otherwise, only minor differences were seen, indicating that the overall whole serum miRNA profiling did not change significantly after 12 weeks of IFN treatment. Next we looked at the dynamic variation of miRNA profiles during (BL, week 12, 24 and EOT) and after therapy (week 24 post-T-FU) according to treatment response (Table 5). NR did not show, by ANOVA analysis, any significant changes throughout the entire observation period. REL revealed one miRNA, let-7b-5p, with significant differential expression over the 5 time-points. SVR during treatment had significant changes in 5 miRNAs, namely miR-122-5p, miR-21-5p, miR-99a-5p, miR-23a-3p and miR-192-5p. MiR-99a-5p and miR-192-5p were always down-regulated on treatment; miR-21-5p was up-regulated at weeks 12 and 24 and EOT, returning to baseline values at Post-T-FU. MiR-23a-3p was up-regulated over the course of treatment and subsequent follow-up. Comparing NR and REL to SVR, at the different time-points, by one-way ANOVA a total of 21 miRNA resulted significantly differentially expressed, after Bonferroni correction (Table 6): miR-320a and miR-320b and miR-335-5p at week 24 during treatment, but not at other time points; miR-99a-5p at EOT and at 24 week post-T-FU. At EOT additional miRNAs, like miR-122-3p and -5p, miR-192-5p and miR-194, were differentially expressed between the groups as compared to post-T-FU, but the differences did not achieve statistical significance after Bonferroni correction.

**Table 4:** Differentially expressed miRNAs comparing Baseline (BL) and Week 12 (Wk 12) sera in 14 Peg-IFN HBeAg negative CHB patients.

Assay	ΔCq (mean ± SD)		ΔΔCq	Student's t test
	BL	Wk 12	Wk 12 - BL	p value
hsa-miR-30e-3p	−3.26±0.51	−1.52±1.35	1.74	0.000354
hsa-let-7b-5p	−0.51±0.27	−0.10±0.31	0.41	0.001731
hsa-miR-27a-3p	−0.56±0.46	−1.06±0.48	−0.50	0.014224
hsa-miR-142-5p	−4.41±0.55	−4.97±0.48	−0.56	0.019861
hsa-miR-29c-3p	−2.34±0.33	−2.02±0.35	0.32	0.027692
hsa-miR-590-5p	−4.01±0.69	−4.72±0.76	−0.71	0.030467
hsa-miR-32-5p	−3.73±0.78	−3.15±0.50	0.57	0.040616
hsa-miR-378a-3p	−1.17±0.65	−1.71±0.57	−0.54	0.042229

Of the 8 miRNAs presenting p<0.05 only miR-30e-3p passes the Bonferroni correction (cut-off <0.000758) for multiple testing (**ΔΔ**Cq 1.7 up-regulation, p = 0.000354).

**Table 5:** Dynamic variation of miRNA profiles at Baseline (BL), during (week 12, 24 and End of Treatment, EOT) and after therapy (week 24 post-treatment follow-up, PT-FU) according to treatment response (NR, REL, SVR) in 14 Peg-IFN treated patients.

		ΔCq (mean ± SD)					ANOVA
Treatment response	Assay	BL	Wk 12	Wk 24	EOT	PT-FU	p-value
**NR**	hsa-miR-24-3p	−0.18±0.25	0.42±0.18	0.47±0.17	0.07±0.33	−0.02±0.12	0.020606
	hsa-miR-335-5p	−3.49±0.29	−2.85±0.36	−3.58±0.27	−3.16±0.23	−2.63±0.52	0.032632
	hsa-miR-103a-3p	−0.07±0.11	−0.24±0.07	0.23±0.26	0.01±0.09	−0.20±0.21	0.035818
**REL**	hsa-let-7b-5p	−0.33±0.23	0.13±0.26	0.16±0.17	−0.19±0.28	−0.49±0.17	0.000054
	hsa-miR-451a	3.15±0.95	4.98±1.50	5.10±1.78	4.38±0.75	2.41±0.91	0.009256
	hsa-miR-92a-3p	2.36±0.55	2.33±0.62	2.39±0.60	1.99±0.21	1.43±0.27	0.022724
	hsa-miR-27a-3p	−0.72±0.33	−1.26±0.37	−1.01±0.52	−0.81±0.53	−0.32±0.32	0.028419
	hsa-miR-24-3p	0.52±0.34	0.08±0.26	0.23±0.17	0.26±0.12	0.13±0.05	0.038510
**SVR**	hsa-miR-122-5p	3.67±1.50	4.75±1.04	3.67±1.33	3.02±0.84	0.96±1.08	0.000001
	hsa-miR-21-5p	2.24±0.41	3.18±0.69	3.22±0.44	3.53±0.45	2.31±0.72	0.000026
	hsa-miR-99a-5p	−0.58±1.39	0.13±0.95	−1.03±1.09	−1.76±0.80	−2.49±0.51	0.000063
	hsa-miR-23a-3p	−0.26±0.50	−0.40±0.59	−0.04±0.54	0.32±0.23	0.55±0.28	0.000083
	hsa-miR-192-5p	−0.30±1.98	0.80±1.05	−0.40±1.57	−0.94±1.34	−2.96±1.46	0.000592
	hsa-miR-27a-3p	−0.31±0.43	−0.76±0.59	−0.61±0.57	−0.31±0.19	0.10±0.37	0.001911
	hsa-miR-23b-3p	−0.59±1.02	−0.74±0.92	−1.58±0.80	−2.04±0.60	−1.65±0.62	0.002195
	hsa-miR-215	−1.79±1.94	−0.63±1.29	−2.15±1.70	−2.93±1.55	−4.27±1.42	0.002369
	hsa-miR-142-3p	1.16±0.69	0.71±0.47	1.15±0.47	1.33±0.38	1.80±0.40	0.002439
	hsa-miR-223-3p	2.05±0.85	1.71±0.87	2.50±0.88	2.64±0.42	3.00±0.45	0.003421

Statistical analysis by one way ANOVA.

**Table 6:** Comparison of miRNA profile of SVR vs NR and REL at different time points by Student’s t test analysis: a total of 21 miRNA (out of 66 tested) showed significant differential expression after Bonferroni correction (cut-off<0.000758).

Assay	ΔCq values (mean±SD) at different time points in treated patients by response														
	Baseline			Wk 12			Wk 24			EOT			PT-FU		
	NR/REL	SVR	p value	NR/REL	SVR	p value	NR/REL	SVR	p value	NR/REL	SVR	p value	NR/REL	SVR	p value
hsa-miR-99a-5p	0.75±0.61	−0.58±1.39	0.021340	0.61±0.49	0.13±0.95	0.243917	0.48±1.14	−1.03±1.09	0.027866	0.58±0.89	−1.76±0.80	0.000271	0.48±0.64	−2.49±0.51	<0.000001
hsa-miR-122-5p	4.83±0.44	3.67±1.50	0.048059	5.20±0.45	4.75±1.04	0.292392	4.84±1.24	3.67±1.33	0.116944	4.98±0.83	3.02±0.84	0.000949	4.62±0.80	0.96±1.08	<0.000001
hsa-miR-194-5p	−0.22±0.43	−1.78±1.88	0.032860	0.17±0.49	−0.90±1.07	0.028206	−0.09±1.23	−2.17±1.88	0.027077	−0.11±1.09	−2.80±1.23	0.001648	−0.40±0.85	−4.22±0.99	0.000001
hsa-miR-192-5p	1.34±0.45	−0.30±1.98	0.033972	1.79±0.59	0.80±1.05	0.044638	1.60±1.15	−0.40±1.57	0.016877	1.66±0.96	−0.94±1.34	0.001802	1.30±0.71	−2.96±1.46	0.000001
hsa-miR-122-3p	0.45±1.05	−1.60±2.33	0.032246	0.45±0.93	−0.60±1.91	0.196555	0.68±1.34	−2.63±2.34	0.005641	0.00±1.04	−3.26±1.95	0.002170	−0.02±0.94	−3.93±1.03	0.000002
hsa-miR-30b-5p	0.10±0.40	−0.45±0.75	0.077031	0.32±0.62	−0.43±0.96	0.100295	0.33±0.80	−0.94±0.96	0.018803	0.22±0.77	−1.35±0.85	0.003482	0.29±0.57	−1.28±0.50	0.000002
hsa-miR-23b-3p	0.25±0.84	−0.59±1.02	0.065659	−0.22±0.55	−0.74±0.92	0.213548	−0.16±0.87	−1.58±0.80	0.008788	−0.43±0.79	−2.04±0.60	0.001333	0.00±0.49	−1.65±0.62	0.000004
hsa-miR-215	0.00±0.68	−1.79±1.94	0.021694	0.26±0.59	−0.63±1.29	0.105766	−0.03±1.27	−2.15±1.70	0.020575	0.13±0.96	−2.93±1.55	0.000983	−0.41±0.87	−4.27±1.42	0.000008
hsa-miR-27b-3p	−0.02±0.52	−0.50±0.76	0.134757	−0.07±0.30	−0.04±0.51	0.903444	−0.06±0.87	−0.76±0.49	0.102072	−0.25±0.61	−0.92±0.43	0.039902	−0.14±0.55	−1.09±0.25	0.000053
hsa-miR-16-5p	0.24±0.94	0.93±0.81	0.087366	0.72±0.78	0.49±0.96	0.632638	0.53±1.04	1.30±0.79	0.155772	0.68±0.88	1.56±0.72	0.069830	0.41±0.71	1.68±0.43	0.000053
hsa-miR-30c-5p	0.39±0.67	−0.47±0.69	0.011765	0.16±0.52	−0.35±1.05	0.247105	0.23±0.91	−0.94±0.93	0.036705	0.02±0.69	−1.34±0.65	0.002911	0.20±0.56	−1.24±0.67	0.000068
hsa-miR-451a	4.21±1.66	5.14±1.55	0.219248	4.77±1.33	4.77±0.91	0.994832	4.27±2.25	5.65±1.16	0.196676	4.40±1.28	5.64±1.05	0.076776	2.98±1.14	5.54±1.12	0.000072
hsa-miR-335-5p	−3.08±0.57	−3.02±0.56	0.824538	−3.29±0.61	−2.85±0.71	0.236619	−3.45±0.43	−2.34±0.28	0.000129	−3.14±0.33	−2.27±0.60	0.004191	−2.66±0.46	−2.72±0.95	0.874613
hsa-miR-23a-3p	−0.63±0.36	−0.26±0.50	0.090519	−1.03±0.70	−0.40±0.59	0.099407	−0.84±0.74	−0.04±0.54	0.045330	−0.70±0.68	0.32±0.23	0.004114	−0.36±0.63	0.55±0.28	0.000189
hsa-miR-320a	1.39±0.52	1.47±0.63	0.768428	1.07±0.45	2.17±0.63	0.002283	0.94±0.44	2.09±0.35	0.000213	1.34±0.55	2.17±0.54	0.015648	0.91±0.45	1.38±0.58	0.070640
hsa-miR-148b-3p	−3.23±0.84	−2.55±0.49	0.028449	−2.81±0.69	−3.08±1.59	0.674942	−3.24±1.02	−2.49±0.72	0.152339	−3.01±0.62	−1.83±0.81	0.009116	−3.17±0.46	−2.02±0.66	0.000410
hsa-miR-148a-3p	0.05±0.52	−1.00±1.23	0.036910	−0.24±0.31	−0.08±0.78	0.590737	−0.23±0.86	−0.47±0.47	0.558978	−0.12±0.79	−0.86±0.62	0.081059	−0.14±0.81	−1.94±0.95	0.000465
hsa-miR-142-5p	−4.57±0.36	−3.58±1.30	0.091416	−5.18±0.44	−4.34±0.50	0.008481	−5.00±0.78	−4.19±0.50	0.051284	−4.93±0.73	−4.31±0.30	0.078933	−4.55±0.68	−2.94±0.84	0.000469
hsa-miR-223-3p	1.49±0.66	2.05±0.85	0.133044	1.06±0.50	1.71±0.87	0.102789	1.29±0.99	2.50±0.88	0.036023	1.32±1.14	2.64±0.42	0.019661	1.76±0.91	3.00±0.45	0.000484
hsa-miR-320b	1.00±0.35	1.15±0.71	0.583403	0.93±0.56	1.75±0.66	0.027984	0.86±0.45	1.87±0.31	0.000487	1.14±0.62	1.97±0.39	0.013767	0.75±0.57	1.20±0.53	0.081259
hsa-miR-484	−1.92±0.48	−1.80±0.57	0.638648	−2.06±0.64	−1.44±0.43	0.066611	−1.90±0.85	−1.15±0.30	0.063498	−1.91±0.59	−1.00±0.35	0.006027	−2.19±0.60	−1.34±0.36	0.000623

#### NUCs treated patients

All HBeAg negative CHB patients treated with NUCs had undetectable HBV-DNA and ALT within the normal range at EOF/post-T-FU evaluation, that was done 59 (33–144) months after treatment start. Comparing the miRNA profiling in baseline and end of follow-up sera we observed an overall reduction in liver specific miRNAs: miR-122, miR-192 and miR-99a (ΔΔCqs of 2.28, 1.77 and 1.35 and p = 0.02, 0.04 and 0.09 respectively). However, only miR-148a-3p showed a significant difference (p = 0.00072) after Bonferroni adjustment.

#### MiR-B-Index, miRNA calculator

We exploited the 3 hepatocellular miRNAs (miR-122-5p+miR-99a-5p+miR-192-5p) with the most significant differential expression in IC versus CHB to build a miRNA calculator. Three additional miRNAs: miR-335 (detected in all serum samples regardless of grouping, and not detected in IP-HBsAg samples), miRNA-126 (down-regulated in serum samples, CHB versus IC, and not detected in IP HBsAg samples) and miR-320a (stable across groups in both serum samples and IP HBsAg) were selected as endogenous controls to account for RNA input variation and other technical variations within the profiling platform. We define this miRNA panel calculator [(Cq miR-122-5p+Cq miR-99a-5p+Cq miR-192-5p)−(Cq miR-126-3p+Cq miR-335-5p+Cq miR-320a)] as MiR-B-Index.

**Figure 2:**
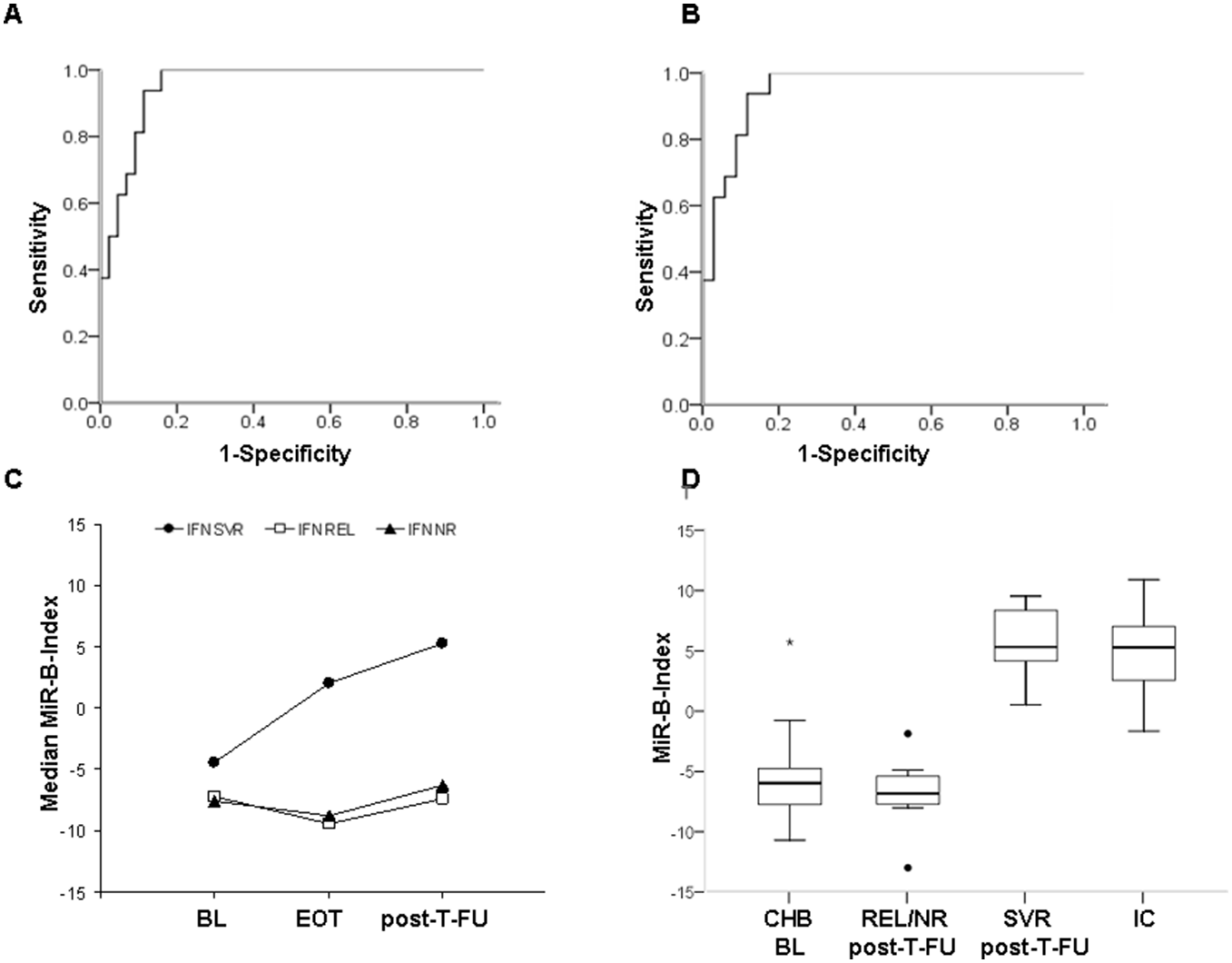
MiR-B-Index in HBV carriers: diagnostic performance to identify inactive carriers (AUROCs), index kinetics in Peg-IFN treated patients by outcome and distribution of the index values by treatment outcome and phase of HBV infection. **A)** Receiver operating characteristic curve for MiR-B-Index in IC vs patients with chronic hepatitis (HBeAg positive or negative CHB, HBV/HDV chronic hepatitis, ALD1): 0.9520 (95% CI 0.903 to 1.000, p<0.001); **B)** ROC for MiR-B-Index in IC vs HBeAg negative CHB (ALD2): 0.954 (95% CI 0.902 to 1.000, p<0.001; **C)** Kinetics of MiR-B-Index in 14 patients treated with Peg-IFN: MiR-B-Index values progressively increased in SVR, whereas they showed minor fluctuations in REL/NR (p<0.001); **D)** Whisker plot of the MiR-B-Index (y-axis) values in BL CHB patients treated with Peg-IFN (NR, REL and SVR), 24 week post-T-FU of REL/NR and SVR and IC, separately (CHB-BL vs IC p<0.001; SVR-BL vs SVR PT-FU p<0.001; REL/NR BL vs REL/NR PT-FU p = 0.462; SVR PT-FU vs IC p = 0.792).

Overall the median MiR-B-Index value in untreated HBV carriers was −4.52 (−16.12/10.91), with significant differences among groups: −15.82 in IT carriers, −13.31 (−16.12/−11.27) in HBeAg positive CHB, −5.38 (−10.71/5.72) in HBeAg negative CHB, −7.36 (−8.85/4.86) in HBV/HDV CH and 5.28 (−1.65/10.91) in IC (p<0.001). Using MiR-B-Index as binary classifier we made a receiver operating characteristic (ROC) curve separating IC versus: a) patients with CHB either HBeAg positive, negative or HBV/HDV [Active Liver Disease group 1 (ALD1); Figure 2A] and b) HBeAg negative CHB [Active Liver Disease group 2 (ALD2); Figure 2B]. The AUROCs of MiR-B-Index to identify IC from ALD1 and ALD2 were 0.9520 (95% CI 0.903–1.000, p<0.001, Figure 2A) and 0.954 (95% CI 0.902–1.000, p<0.001, Figure 2B), respectively. By using a cut-off value of −1.7 while classifying IC from ALD1 and ALD2, 100% sensitivity, 84.4 and 83.3% specificity, 69.6% and 72.7% PPVs, 100% NPVs, 88.5% diagnostic accuracy (DA) were achieved.

In the 21 Peg-IFN treated patients the median BL and post-T-FU MiR-B-Index values were significantly different [−5.81 (−10.71/5.72) and 3.76 (−12.99/9.53) respectively, p = 0.022]. At BL MiR-B-Index values were higher in SVR (−4.49, −10.71/5.72) as compared to REL (−7.23, −9.99/−5.81) and NR (−7.56, −7.93/−7.26), p = 0.047: the difference resulted from the MiR-B-Index values (median 3.70, −0.77/5.72) observed in 4 patients, whose HBV-DNA (4.45, 2.67–5.19 Log_10_ IU/mL) and HBsAg (2.48, 1.13–3.54 Log_10_ IU/mL) levels were lower than in the remaining 8 (HBV-DNA: 5.83, 0.78–7.42 Log_10_ IU/mL; HBsAg 3.5, 2.87–4.09 Log_10_ IU/mL, p = 0.123 and p = 0.045, respectively) with MiR-B-Index (−5.03, −10.71/−3.27) comparable to REL/NR (−7.41, −9.99/−5.81). At 24 week post-T-FU in SVR MiR-B-Index values were significantly higher (5.32, 0.54/9.53) as compared to REL (−7.41, −12.99/−1.87) and NR (−6.31, −7.37/−5.92), p = 0.001: all SVR had values above the −1.7 MiR-B-Index cut-off (Figure 2C–D). On treatment MiR-B-Index kinetics were studied in 14 patients: at EOT MiR-B-Index increase was observed in all SVR (2.02, −2.06/5.84), 4 with values >−1.7 (Figure 2C), but not in REL and NR (−9.49, −11.65/−3.58 and −8.82, −12.17/−3.99, respectively), p = 0.008.

Overall in the 11 NUCs treated patients MiR-B-Index showed a trend to improve [BL vs post-T-FU median values −5.86 (−13.39/6.57) vs 0.12 (−8.41/9.46), p = 0.071]. The MiR-B-Index increase did not correlate with treatment duration (*ρ* = 0.127, p = 0.709). In 5 patients whose MiR-B-Index increased above the −1.7 cut-off antiviral therapy was discontinued because of HBsAg/anti-HBs seroconversion in 2 while serum HBsAg levels dropped below 400 IU/mL (14.6, 300 and 380 IU/mL) in the remaining 3 who continued treatment. The MiR-B-Index of the 2 NUC-treated patients with HBsAg/anti-HBs seroconversion (9.46 and 7.08) were comparable with IFN-SVRs at post-T-FU (5.32, 0.54/9.53).

#### Validation of MiR-B-Index

The MiR-B-Index was retested in 15 samples of the training cohort using single microRNA specific RT-q-PCR and showed values highly comparable with those obtained by Ready to use micro-RNA-PCR panel I/II (data not shown). In untreated HBV carriers the median MiR-B-Index values were −6.07 (−20.43/14.38), with significant variation between groups: −11.17 (−20.43/−7.73) in IT, −14.20 (−18.06/−4.63) in HBeAg positive CHB, −6.82 (−18.42/−0.18) in HBeAg negative CHB and 7.29 (−1.41/14.38) in IC (p<0.001). We ran a ROC curve analysis to discriminate IC from HBeAg negative CHB and the AUROC value was 0.995 (95% CI 0.984–1.000; p<0.000001). The MiR-B-Index values ≥−1.7 identified IC with 100% sensitivity, 92.5% specificity, 88.5% PPV, 100% NPV and 95.2% diagnostic accuracy. In the 15 Peg-IFN treated patients (9 HBeAg positive) the MiR-B-Index at 24 week post-T-FU was significantly different from BL [−4.24 (−16.80/5.94) vs −14.20 (−18.06/−7.25), p<0.001) and showed significantly higher values in SVR patients as compared to REL/NR [2.9 (0.07/5.94) vs −7.04 (−16.80/−4.09), p = 0.001). Overall, MiR-B-Index increased in the 37 NUCs treated patients [BL vs post-T-FU median values −6.73 (−18.42/−0.33) vs 0.07 (−9.54/4.17), p<0.000001].

#### MiR-B-Index and ALT, HBV-DNA and HBsAg serum levels

Because the MiR-B-Index performance was highly comparable in both training and validation cohorts, the correlation between its values and ALT, HBV-DNA and HBsAg serum levels were performed combining the 2 cohorts.

Overall at BL MiR-B-Index (−5.72, −20.43/14.38) showed a significant correlation with ALT (49, 10/2056 U/l, *ρ* = −0.497, p<0.001), HBV-DNA (4.58, undetectable/>8.3 Log_10_ IU/mL, *ρ* = −0.732, p<0.001) and HBsAg serum levels (3.40, 0.11–5.49 Log_10_ IU/mL, *ρ* = −0.883, p<0.001). When we considered ALT values by class (≤40, 41–80, 81–120, >120 U/L) we found that MiR-B-Index values in patients with ALT ≤40 U/L were significantly higher than in the others (p<0.000001) whereas they were similar in the 3 groups with ALT>40 U/L [−7.23 (−20.43/6.57) vs −8.64 (−18.42/−2.21) vs −6.59 (−16.99/4.86), p = 0.102]. Immune Tolerant carriers in spite of normal ALT as Inactive Carriers (26, 19–41 U/L vs 20, 10–42 U/L), had MiR-B-Index values significantly different from IC [−11.61 (−20.43/−7.73) vs 6.68 (−1.65/14.38), p<0.001], but comparable to those of HBeAg positive CHB patients [−14.47 (−18.06/−4.63), p = 1.00]. At multivariate analysis factors independently associated with MiR-B-Index were HBV-DNA (p = 0.002) and HBsAg (p<0.001) serum levels, phase of HBV infection (IC, IT, HBV/HDV, HBeAg positive and HBeAg negative CHB; p<0.001), but not ALT values (p = 0.360).

HBsAg serum levels were significantly correlated with MiR-B-Index in untreated carriers [IC, IT, HBeAg-positive and negative CHB at BL: HBsAg 3.38, 0.11–5.49 Log_10_ IU/mL and MiR-B-Index −5.61 (−20.43/14.38); *ρ* = −0.904, p<0.001), but not in the post-T-FU sera of IFN-SVR patients (HBsAg 1.29, undetectable/2.70 Log_10_ IU/mL; MiR-B-Index 4.96, 0.07/9.53; *ρ* = −0.423, p = 0.08). In NUC-treated patients MiR-B-Index increased significantly from BL to EOF independently of the extent of HBsAg decline [BL and EOF MiR-B-Index values in patients with >1 log HBsAg decline −6.56 (−18.42/0.37) vs 1.99 (−3.46/14.17), p<0.001; BL and EOF MiR-B-Index values in patients with <1 log HBsAg decline −6.62 (−15.18/6.57) vs −1.25 (−9.54/9.22), p<0.001]. The MiR-B-Index Δ-variation from BL to EOF was significantly higher in patients with HBsAg decline >1 log [12.56 (4.88/19.39) vs 5.03 (−5.97/15.95), p<0.001) and associated with BL MiR-B-Index values (ρ = −0.450, p = 0.001), NUC treatment duration (ρ = 0.310, p = 0.032), BL HBsAg serum levels (ρ = −0.320, p = 0.039) and HBsAg Δ-variation from BL to EOF (ρ = −0.663, p<0.0001), but not with BL ALT and HBV-DNA levels. At multivariate analysis only BL MiR-B-Index values (p = 0.007) and HBsAg Δ-variation between BL and EOF (p = 0.002) resulted independently associated with the Δ-decline of MiR-B-Index.

## Discussion

The study of serum miRNA of well characterized HBsAg carriers shows important variations of their profiles across the different phases of chronic HBV infection: the major difference is observed between inactive carriers and chronic hepatitis patients, who show a highly significant over-expression of miR-122-5p, miR-99a-5p and miR-192-5p (Table 3). The same miRNAs reveal a consonant profile in circulating HBsAg-particles, where major differences in the expression of the 32 most commonly detected miRNAs, were observed when comparing IC to untreated CHB patients. In patients with SVR to Peg-IFN the post-T-FU sera presented miRNA profiles highly similar to IC (Table 2) and the heatmap demonstrated the clustering of these 2 groups (IC and SVR-post-T-FU samples; Figure 1). On the contrary, in Relapsers and Non Responders the post-T-FU miRNAs patterns matched those of BL. These findings support the hypothesis that consistent changes in circulating miRNA profiles parallel the immune control of HBV infection. Accordingly, CHB patients with SVR to Peg-IFN, at variance with REL and NR, experienced the largest difference in their serum miRNA signature overtime, with major variations of the miRNA average signal (ΔCq) when comparing post-T-FU with BL samples (Table 2; Figure 1). In 14 patients whose miRNA profiles could be studied across their whole treatment course we observed that serum miRNA patterns did not change significantly during the first 12 weeks of Peg-IFN, with the exception of miR-30e-3p, that was up-regulated in all patients (Table 4; p = 0.000354). Our finding is in agreement with a previous report suggesting an early induction of the miRs-30 cluster by IFN independently of therapy response [26]. During the course of treatment, NR patients did not show any significant variation of additional miRNAs, whereas REL had significant differential expression over the 5 time-points of miRNA let-7b-5p (Table 5). The implications of such miRNA modulation deserve further investigation since the up-regulation of let-7 family during IFN therapy was reported to inhibit hepatitis C virus replication [29]. The comparison of NR/REL and SVR at every time point (Table 6) showed that 21 miRNAs were differentially expressed and 5 of them, miR-122-5p, miR-21-5p, miR-23a-3p, miR-99a-5p and miR-192-5p had the most significant changes during treatment in SVR. MiRNA-99a-5p and miRNA-192-5p were down-regulated throughout treatment and post-T-FU, whereas miRNA-21-5p was up-regulated at week 12, 24 and EOT and returned to BL-values at post-T-FU. Interestingly miR-122-5p, miR-99a-5p and miR-192-5p showed parallel differential patterns in whole serum and HBsAg-particles of IC and CHB patients once they achieved SVR as compared to untreated CHB and REL/NR. These 3 miRNAs are the 1^st^, 2^nd^ and 6^th^ most represented miRNAs of human liver [16], [28]: serum miR-122 and miR-99 levels had been shown to be higher in HBsAg carriers than healthy controls [16] and miR-122 and miR-192 associated with liver necro-inflammation [30]–[34].

The findings prompted us to evaluate a combination of the most significant miRNAs to produce a MiR-B-Index to identify the sustained switch from active to inactive HBV infection in treated patients with CHB and eventually to improve the inactive-carrier diagnostic accuracy. We combined the 3 liver-miRNAs with miR-335, miR-126 and miR-320a for internal normalization and, by using a cut-off of −1.7, the MiR-B-Index showed a high diagnostic performance both in the training (100% sensitivity, 83.3% specificity, 72.7% PPV, 100% NPV, 88.5% DA) and in the validation (100% sensitivity, 92.5% specificity, 88.5% PPV, 100% NPV and 95.2% DA) cohorts in identifying IC from CHB. In addition, all patients who achieved off-therapy SVR either after Peg-IFN or NUCs showed significant MiR-B-Index improvements during therapy with values at their post treatment follow-up highly comparable to those of IC (5.32 vs 6.68, p = 0.324). All these evidences propose the MiR-B-Index as a candidate biomarker to identify either spontaneous or therapy induced transition from active to inactive phase of chronic HBV infection. Accordingly, MiR-B-Index showed a significant correlation with those parameters (ALT, HBV-DNA and HBsAg; p<0.001 for all), that are currently used to manage HBV carriers, however it appears to provide additional information, contributing to a more stringent characterization at the single patient level. First of all, MiR-B-Index values in spite of being associated with ALT levels, resulted to be primarily influenced by the phase of HBV infection (p<0.001): in fact, HBeAg positive immune-tolerant carriers, in spite of their normal ALT, had MiR-B-Index values comparable to those of HBeAg positive CHB patients. Furthermore, during NUCs treatment even though all the patients normalized ALT, only a proportion of them experienced a significant improvement of MiR-B Index. These findings do not support the hypothesis that the MiR-B-Index results from the amount of liver damage, but suggest that it could indeed mirror the extent of HBV infection control. Indeed in untreated HBV carriers its values showed a very high correlation (ρ = −0.904, p<0.001) with HBsAg serum levels, that are inversely correlated with the extent of the immune control [6], [8]. All NUC treated patients with HBsAg/anti-HBs seroconversion or decline of HBsAg serum levels <400 IU/mL showed a consistent MiR-B-Index improvement with values above the −1.7 cut-off. Overall, MiR-B-Index variations during NUC therapy appear closely linked with those of HBsAg, however our findings suggest that its variations beckon the effective achievement of sustained immune control of HBV with faster kinetics than HBsAg [5]–[6]. Accordingly, in SVR to Peg-IFN the correlation between MiR-B-Index and HBsAg serum levels at post-T-FU was not significant (ρ = −0.423, p = 0.08) and among the 6 patients with SVR in whom we tested EOT samples the MiR-B-Index was already improved with IC like values in 4 (66%). Thus, it appears that, during antiviral therapy, MiR-B-Index provides complementary information to HBsAg monitoring.

Furthermore, a few HBeAg negative CHB patients with SVR to Peg-IFN showed MiR-B-Index values comparable to IC already at baseline with further improvement during treatment, whereas this never occurred in NR and REL; during NUC therapy, the improvement of MiR-B-Index was influenced by baseline MiR-B-Index values. Both evidences suggest that MiR-B-Index could help to identify patients susceptible to respond to Peg-IFN and to achieve a sustained control of HBV infection during a time limited NUC treatment. Altogether these findings propose the MiR-B-Index as candidate biomarker to predict and to identify either spontaneous or therapy induced transition from active to inactive phase of chronic HBV infection. MiR-B-Index might be helpful to tailor antiviral treatment at the individual level, identifying NUCs treated patients who could stop therapy safely without risk of hepatitis B relapse once they achieve a sustained immune control of HBV infection or IFN treated patients who could benefit from prolonged treatment.

In conclusion our study shows for the 1^st^ time that the dynamic change of a miRNA signature may identify both natural occurring and therapy induced immune control of HBV infection. The same signature qualifies as new diagnostic biomarker to satisfy the unmet need of the early identification of the sustained switch from chronic active hepatitis B to the inactive HBV infection in patients treated with antivirals. MiR-B-Index is worth being tested in larger cohorts of patients infected with different HBV genotypes, treated with different antivirals and with different therapy outcomes.
